# Oxidative stress impairs cognitive function by affecting hippocampal fimbria volume in drug-naïve, first-episode schizophrenia

**DOI:** 10.3389/fnins.2023.1153439

**Published:** 2023-04-17

**Authors:** Rufei Jia, Xiuxia Yuan, Xiaoyun Zhang, Peilun Song, Shaoqiang Han, Shuying Wang, Yajun Li, Siwei Zhang, Xinyi Zhao, Yu Zhang, Jingliang Cheng, Xueqin Song

**Affiliations:** ^1^Department of Psychiatry, The First Affiliated Hospital of Zhengzhou University, Zhengzhou, China; ^2^Biological Psychiatry International Joint Laboratory of Henan, Zhengzhou University, Zhengzhou, China; ^3^Henan Psychiatric Transformation Research Key Laboratory, Zhengzhou University, Zhengzhou, China; ^4^School of Information Engineering, Zhengzhou University, Zhengzhou, China; ^5^Department of Magnetic Resonance Imaging, The First Affiliated Hospital of Zhengzhou University, Zhengzhou, China

**Keywords:** schizophrenia, oxidative stress, hippocampal subfields, cognitive impairments, hippocampal fimbria volume

## Abstract

**Objective:**

The aim of the present study was to explore influencing factors of cognitive impairments and their interrelationships in drug-naïve, first-episode schizophrenia (SCZ).

**Methods:**

Patients with drug naïve, first episode SCZ and healthy controls (HCs) were enrolled. Cognitive function was assessed by the MATRICS Consensus Cognitive Battery (MCCB). Serum levels of oxidative stress indices, including folate, superoxide dismutase (SOD), uric acid (UA) and homocysteine (Hcy), were determined after an overnight fast. Hippocampal subfield volumes were measured using FreeSurfer. Mediation models were conducted using the SPSS PROCESS v3.4 macro. A false discovery rate (FDR) correction was applied for multiple comparisons.

**Results:**

Sixty-seven patients with SCZ and 65 HCs were enrolled in our study. The patient group had significantly lower serum levels of folate and SOD and higher serum levels of HCY compared with the HCs (all *p* < 0.05). The patient group had a significantly smaller volume of the whole hippocampus than the HC group (*p* < 0.05). We also found significant volume differences between the two groups in the following subfields: CA1, molecular layer, GC-ML-DG and fimbria (all *p* < 0.05, uncorrected). The partial correlation analysis controlling for age and sex showed that the fimbria volume in the patient group was significantly positively associated with NAB scores (*r* = 0.382, pFDR = 0.024); serum levels of SOD in the patient group showed a significantly positive correlation with fimbria volume (*r* = 0.360, pFDR = 0.036). Mediation analyses controlling for age and sex showed that the serum levels of SOD in patients with SCZ had significant indirect effects on the NAB scores which were mediated by the fimbria volume [indirect effect = 0.0565, 95% CI from the bootstrap test excluding zero (0.0066 to 0.0891)].

**Conclusion:**

Oxidative stress, a reduction in hippocampal subfield volumes and cognitive impairments occur in early SCZ. Oxidative stress impairs cognitive function by affecting hippocampal subfield volumes.

## Introduction

Schizophrenia (SCZ) is a common and severe mental disorder characterized by positive symptoms, negative symptoms and cognitive impairments. With a prevalence of nearly 1%, SCZ is one of the top 10 causes of disability worldwide (Marder and Cannon, 2019). Atypical antipsychotics can improve positive and negative symptoms but are less effective in treating cognitive symptoms. Cognitive impairments are stable and persistent in patients with SCZ and severely affect the social functions of patients. The potential pathophysiological mechanisms of cognitive deficits in SCZ remain unclear. There is growing evidence that hippocampal dysfunction and oxidative stress are associated with impaired cognitive function in patients with SCZ (Maas et al., 2017; Antoniades et al., 2018; Tao et al., 2020; Duan et al., 2021).

Robust hippocampal volume deficits are commonly reported in SCZ (Van Erp et al., 2016). The hippocampus has been associated with complex cognitive functions, including working memory (Hahn et al., 2012), verbal learning (Nikolova et al., 2017), visual memory (Postma et al., 2020) and spatial navigation (Ruediger et al., 2012). However, the hippocampus is a heterogeneous structure that consists of subregions with distinct functions (Zeidman and Maguire, 2016). Several studies have reported the following abnormalities in SCZ: hyperactivity in cornu ammonis (CA) 1 (Lander et al., 2019), GABAergic abnormalities in CA2/3 (Benes, 2015) and hypoglutamatergic activity in the dentate gyrus (DG) (Jiao et al., 2017). These findings indicate that hippocampal subfields may play different roles in the pathophysiology of SCZ. Hippocampal storage and recall are closely related to the subtle structure of different subregions. The hippocampal DG-CA3 circuit is responsible for storing memories, while the CA1, CA3, and subiculum are necessary for recalling memories (O'Reilly and McClelland, 1994). Two previous studies found that different hippocampal subregions were involved in different cognitive domains in SCZ (Vargas et al., 2018; Nakahara et al., 2020). The reasons underlying the volume reductions in the hippocampus and its subregions in SCZ are still unclear.

Oxidative stress occurs as a result of increased free radicals and decreased antioxidants. Many studies have shown that oxidative stress is involved in the pathophysiology of SCZ (Hardingham and Do, 2016; Alameda et al., 2018; Pistis et al., 2022). Reactive oxygen species (ROS) are the most important class of free radicals in living organisms. A slight increase in ROS levels promotes the proliferation and differentiation of cells. However, excessive ROS can damage DNA, proteins and lipids through oxidation (Wu et al., 2013; Romano et al., 2017). Polyunsaturated fatty acids (PUFAs) in cell membranes are highly susceptible to oxidative insult. High levels of PUFAs in brain tissue, combined with high oxygen consumption, make brain structures particularly vulnerable to oxidative damage (Mitra et al., 2017). It can be hypothesized that hippocampal volume loss may be influenced by oxidative stress to a large extent. According to this hypothesis, decreased DG-CA4 volume was shown to be associated with blood measures of oxidative stress in bipolar disorders (Elvsåshagen et al., 2016). Reduced hippocampus in SCZ linked to neuronal atrophy and loss of neuropil, which may be the result of redox dysregulation (Harrison, 2004). Hippocampal volumetric integrity in patients with SCZ was associated with several peripheral biomarkers of oxidative stress including glutathione peroxidase (GPx) and S100 calcium binding protein B (S100B) (Baumann et al., 2016; Goff et al., 2018). These suggest that peripheral markers of oxidative stress may reflect alterations in the brain. Whether peripheral indicators of oxidative stress are associated with hippocampal subfield volumes in patients with SCZ has not yet been reported.

ROS have an extremely short half-life and are difficult to examine *in vivo*. As a key antioxidant enzyme, superoxide dismutase (SOD) can detoxify superoxide radicals and prevent lipid peroxidation. Peripheral levels of SOD were significantly lower in patients with first-episode psychosis but higher in chronic patients versus healthy controls (Chen et al., 2022). An animal model with SCZ induced by ketamine showed decreased levels of SOD and catalase in the brain. Inhibition of the kynurenine pathway could prevent oxidative stress (Réus et al., 2018). Folate, an important antioxidant, was found to reduce the levels of superoxide anion through nicotinamide adenine dinucleotide phosphate (NADPH) oxidase (Hwang et al., 2011). Homocysteine (Hcy) can induce oxidative stress by increasing the intracellular concentration of ROS and disrupting antioxidant systems (Esse et al., 2019). Moreover, an increase in HCY or folate deficiency can result in methylation alterations and/or redox imbalance (Cordaro et al., 2021). Methylation is critical to brain development and function (Menezo et al., 2022). Uric acid (UA) is an antioxidant, and lower UA levels can cause increased oxidative stress and degeneration of dopamine neurons (Seifar et al., 2022). Our previous results showed that the serum levels of oxidative stress were significantly correlated with cognitive function in subjects with SCZ (Tao et al., 2020). The exact mechanism explaining the association between oxidative stress indicators and cognitive function in patients with SCZ is not clearly understood.

The present study aimed to explore influencing factors of cognitive impairments and their interrelationships in drug-naïve, first-episode schizophrenia (SCZ). We hypothesized that hippocampal subregion volumes are associated with cognitive function and oxidative stress and that oxidative stress impairs cognitive function by affecting hippocampal subregion volumes in the patient group.

## Materials and methods

### Subjects

This study was approved by the Ethics Committee of the First Affiliated Hospital of Zhengzhou University. All subjects with SCZ were recruited from inpatient populations. The inclusion criteria for the patient group included the following: (1) diagnosis of first-episode SCZ based on the Diagnostic and Statistical Manual of Mental Disorders fourth version (DSM-IV) criteria and confirmed using the Structured Clinical Interview for DSM-IV; (2) 18-45 years of age; (3) no use of antipsychotics or other psychotropics; (4) a total PANSS score ≥ 60; and (5) duration of the disease < 5 years. The exclusion criteria included (1) a diagnosis of diabetes, autoimmune diseases, neurological or other mental disorders, heart diseases, blood diseases, endocrine system diseases and other organic diseases; (2) a history of head injury or substance abuse; (3) pregnancy; and (4) treatment with folate supplements or antioxidants. Healthy controls (HCs) were recruited from the local community through advertisements. The exclusion criteria for HCs were the same as those for the patient group. None of the HCs had a history of any mental illness. Written informed consent was obtained from all subjects.

### Assessments

The severity of symptoms was assessed in all patients using the Positive and Negative Syndrome Scale (PANSS). The PANSS was performed by an experienced psychiatrist. Cognitive function was assessed for all subjects by using the MATRICS Consensus Cognitive Battery (MCCB), which consists of nine subtests covering the following cognitive domains: (1) Trail Making Test, Part A (TMT-A); (2) Brief Assessment of Cognition in Schizophrenia, Symbol Coding subtest (BACS-SC); (3) Hopkins Verbal Learning Test-Revised (HVLT-R); (4) Wechsler Memory Scale, 3rd edition (WMS-III); (5) Neuropsychological Assessment Battery (NAB); (6) Brief Visuospatial Memory Test-Revised (BVMT-R); (7) Category Fluency; (8) Mayer-Salovey-Caruso Emotional Intelligence Test (MSCEIT); and (9) Continuous Performance Test-Identical Pairs (CPT-IP). The Speed of Processing domain (SOP) was assessed with TMT-A, BACS-SC and category fluency.

### Laboratory tests

After an overnight fast, venous blood samples were collected between 6:30 am and 7:30 am on the second day after admission. Whole blood was placed in EDTA anticoagulation tubes and centrifuged at 3000 rpm for 10 min to obtain the serum. The serum levels of folate were determined by electrochemiluminescence immunoassay (Abbott Laboratories, United States). The serum levels of SOD were determined by kits and a Roche automatic biochemical analyzer (Roche Diagnostics, C8000, Germany). The serum levels of Hcy were determined by the enzymatic cycling method (Kangte Bio-Tech, China). The serum levels of UA were determined by the uricase-peroxidase method (Roche, C720, Switzerland). The assays were performed by a medical laboratory technician who was blinded to the subjects’ diagnosis.

### MRI acquisition and processing

**Figure 1:**
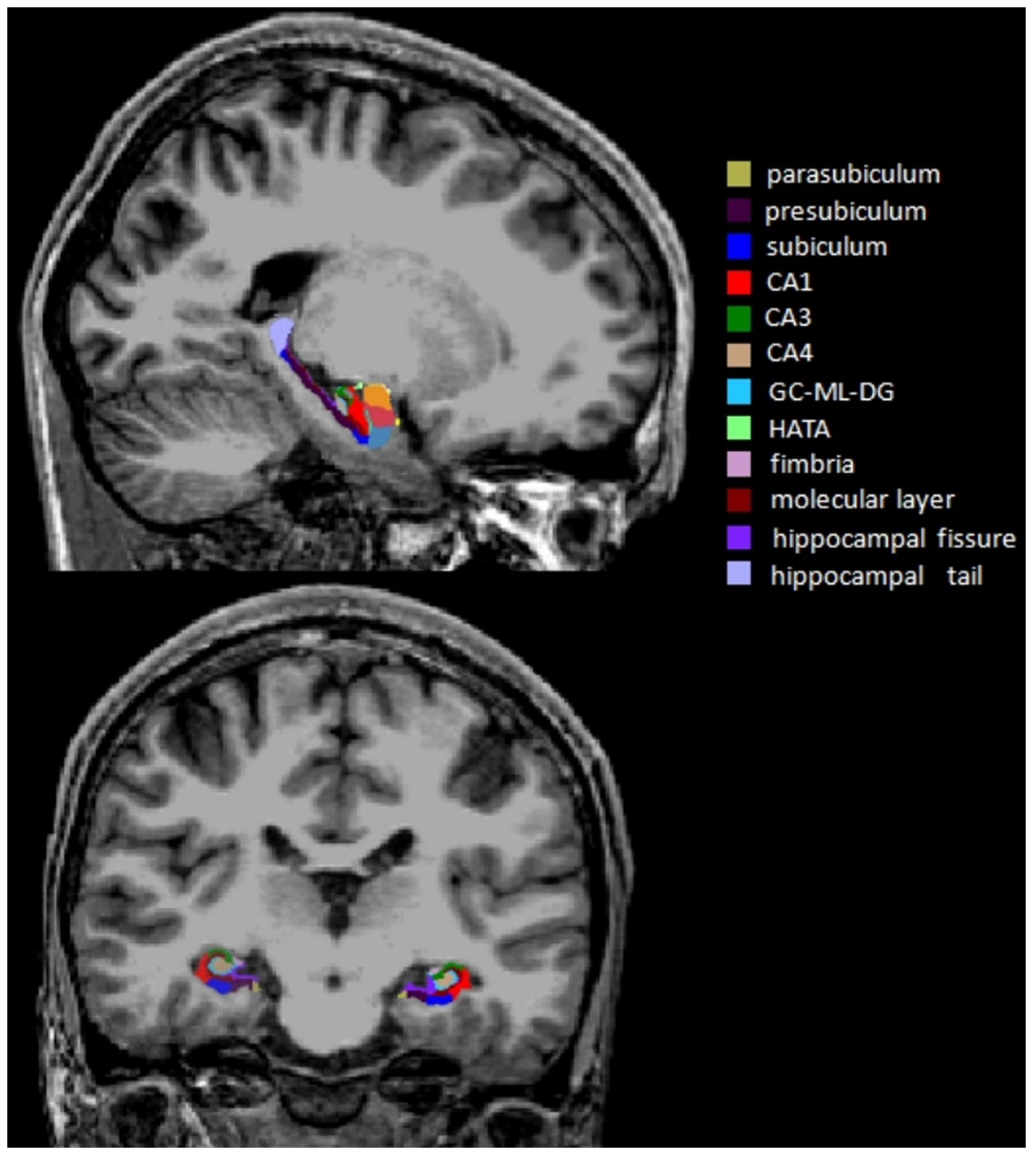
Sagittal and coronal views of the hippocampal subfield segmentation from a single subject. CA, cornu ammonis; GC-ML-DG, granule cells in the molecular layer of the dentate gyrus; HATA, hippocampal-amygdaloid transition area.

MRI data were obtained with a 3.0 Tesla Scanner (GE Discovery MR750 3.0 T) at the First Affiliated Hospital of Zhengzhou University. High-resolution T1-weighted images were acquired using the following parameters: repetition time (TR) = 8.2 ms, echo time (TE) = 3.2 ms, flip angle = 12°, field of view (FOV) = 256 × 256 mm2, matrix size = 256 × 256, slice thickness = 1 mm, slice number = 188, and slice gap = 0 mm. During the scan, all subjects were required to relax, hold still and keep their eyes closed. All images were visually inspected to eliminate images with motion or metal artifacts. No images were removed. FreeSurfer software (version 7.1^1^) was used to automatically estimate whole hippocampal volume, hippocampal subfield volumes and total intracranial volume (ICV). The major processing operations of FreeSurfer included head motion correction, automated Talairach transformation, intensity normalization, skull stripping, segmentation of the cortical and subcortical gray and white matter structures, surface reconstruction, registration and parcellation. Twelve subfield volumes were assessed by the subfield segmentation protocol (v7.1) in each hemisphere: hippocampal tail, presubiculum, parasubiculum, subiculum, hippocampal fissure, CA1, CA3, CA4, molecular layer, GC-ML-DG, fimbria and HATA (Iglesias et al., 2015; Figure 1).

### Statistical analysis

IBM SPSS Statistics 26.0 software was used for data analysis. Whole hippocampal and subfield volumes were corrected for ICV via the covariance method: Volume (adjusted) = Volume (observed) -B(TCV_i_-TCV_mean_), where TCV_i_ = the subject’s total intracranial volume, TCV_mean_ = overall average total intracranial volume, and B is the slope of the regression line of hippocampal volume regressed on total intracranial volume (Jack et al., 1989). The mean volumes across hemispheres were used in the following analyzes to reduce the multiple testing burden and increase statistical power. Group comparisons were performed using the independent sample t test or Mann–Whitney U test for continuous variables and χ2 test for categorical variables. Partial correlation was used to examine relationships between oxidative stress, cognitive function and hippocampal subfield volumes controlling for age and sex. Mediation analyzes were performed using the PROCESS v3.4 macro. Significant indirect effects were indicated when the 95% confidence interval of 5,000 bootstrap realizations did not include zero. A false discovery rate (FDR) correction was applied for multiple comparisons. The statistical significance was set at two-tailed *p* < 0.05.

## Results

### Demographic and clinical data

Sixty-seven patients with SCZ and 65 healthy controls were enrolled in our study. There were no significant differences in the age, sex, education or body mass index (BMI) between the two groups (all *p* > 0.05; Table 1). The patient group had significantly lower serum levels of folate and SOD and higher serum levels of Hcy than the controls (*p* < 0.05, *p* < 0.001, *p* < 0.001, respectively; Table 1). There was no significant difference in serum levels of UA between the two groups (*p* > 0.05). The patients had significantly lower scores in the seven domains of cognitive function than the controls (All p < 0.05; Table 1).

**Table 1:** Demographic and clinical characteristics of participants.

Characteristics	Patients (*n* = 67)	Healthy controls (*n* = 65)	*t*/*Z*/*χ*^2^	*p*
Age(years)	23.55 ± 6.07	23.14 ± 1.65	−1.621	0.105
Sex(male/female)	33/34	22/43	3.222	0.073
Education (years)	11.84 ± 2.33	12.38 ± 0.91	−1.795	0.073
BMI (kg/m^2^)	20.78 ± 10.34	20.85 ± 2.57	−0.241	0.809
Folate(ng/ml)	7.38 ± 3.42	9.49 ± 4.56	−2.579	0.01*
SOD(U/ml)	179.96 ± 29.93	206.48 ± 32.10	−6.253	<0.001**
UA(μmol/L)	292.06 ± 91.20	268.15 ± 69.96	−1.202	0.229
Hcy(μmol/L)	21.78 ± 15.97	12.65 ± 7.36	−5.783	<0.001**
Disease duration (months)	15.57 ± 20.78	–	–	–
PANSS				
Positive symptoms	21.48 ± 4.60	–	–	–
Negative symptoms	22.84 ± 6.52	–	–	–
General psychopathology	45.28 ± 8.22	–	–	–
Total scores	89.60 ± 15.50	–	–	–
Cognitive function				
SOP	28.12 ± 14.25	47.72 ± 7.54	−9.922	<0.001**
CPT-IP	29.87 ± 13.66	49.05 ± 9.51	−9.386	<0.001**
WMS-III	39.15 ± 11.18	49.45 ± 9.89	−5.114	<0.001**
HVLT-R	35.46 ± 10.93	45.60 ± 8.76	−5.484	<0.001**
BVMT-R	38.87 ± 15.89	45.72 ± 9.89	−2.405	0.016*
NAB	34.87 ± 10.52	39.20 ± 8.80	−2.564	<0.001**
MSCEIT	33.84 ± 12.01	42.29 ± 9.16	−4.85	<0.001**

BMI, body mass index; SOD, superoxide dismutase; UA, uric acid; Hcy, homocysteine; PANSS, Positive and Negative Syndrome Scale; SOP, Speed of Processing; CPT-IP, Continuous Performance Test-Identical Pairs; WMS-III, Wechsler Memory Scale, 3rd edition; HVLT-R, Hopkins Verbal Learning Test-Revised; BVMT-R, Brief Visuospatial Memory Test-Revised; NAB, Neuropsychological Assessment Battery; MSCEIT, Mayer-Salovey-Caruso Emotional Intelligence Test. *p < 0.05; **p < 0.01.

### Group differences in hippocampal subfield volumes

The SCZ patients had a significantly smaller volume of the whole hippocampus compared to the controls (*p* < 0.05; Table 2). We also found significant volume differences between the two groups in the following subfields: CA1, molecular layer, GC-ML-DG and fimbria (CA1:*t* = −2.185, *p* = 0.031, uncorrected; molecular layer: *t* = −2.39, *p* = 0.018, uncorrected; GC-ML-DG: *Z* = −2.132, *p* = 0.033, uncorrected; fimbria: *Z* = −2.274, *p* = 0.023, uncorrected). There were no significant differences in other hippocampal subfield volumes between the two groups (All *p* > 0.05).

**Table 2:** Differences in hippocampal subfield volumes (mm^3^) between patients with schizophrenia and healthy controls.

	Patients (*n* = 67)	Healthy controls (*n* = 65)	*t*/*Z*	*p*
Whole hippocampus	3826.67 ± 425.94	3959.60 ± 309.67	−2.055	0.042*
Hippocampal tail	658.80 ± 85.63	676.83 ± 91.08	−1.172	0.243
Presubiculum	346.07 ± 50.18	354.52 ± 45.15	−1.268	0.205
Parasubiculum	69.22 ± 13.29	68.78 ± 10.91	0.206	0.837
Subiculum	487.95 ± 59.21	503.35 ± 48.27	−1.791	0.073
Hippocampal fissure	163.09 ± 29.13	158.34 ± 22.64	1.043	0.299
CA1	702.86 ± 83.41	731.76 ± 67.45	−2.185	0.031*
CA3	219.20 ± 30.76	228.25 ± 27.42	−1.919	0.055
CA4	264.54 ± 33.18	273.52 ± 24.15	−1.782	0.077
Molecular layer	609.07 ± 68.33	634.28 ± 52.02	−2.39	0.018*
GC-ML-DG	310.84 ± 39.37	322.32 ± 27.66	−2.132	0.033*
Fimbria	93.32 ± 19.95	100.33 ± 21.28	−2.274	0.023*
HATA	64.83 ± 9.33	65.64 ± 7.15	−1.113	0.266

CA, cornu ammonis; GC-ML-DG, granule cells in the molecular layer of the dentate gyrus; HATA, hippocampal-amygdaloid transition area. * *p* < 0.05.

### Correlations among oxidative stress, hippocampal subfield volumes and cognitive function

**Figure 2:**
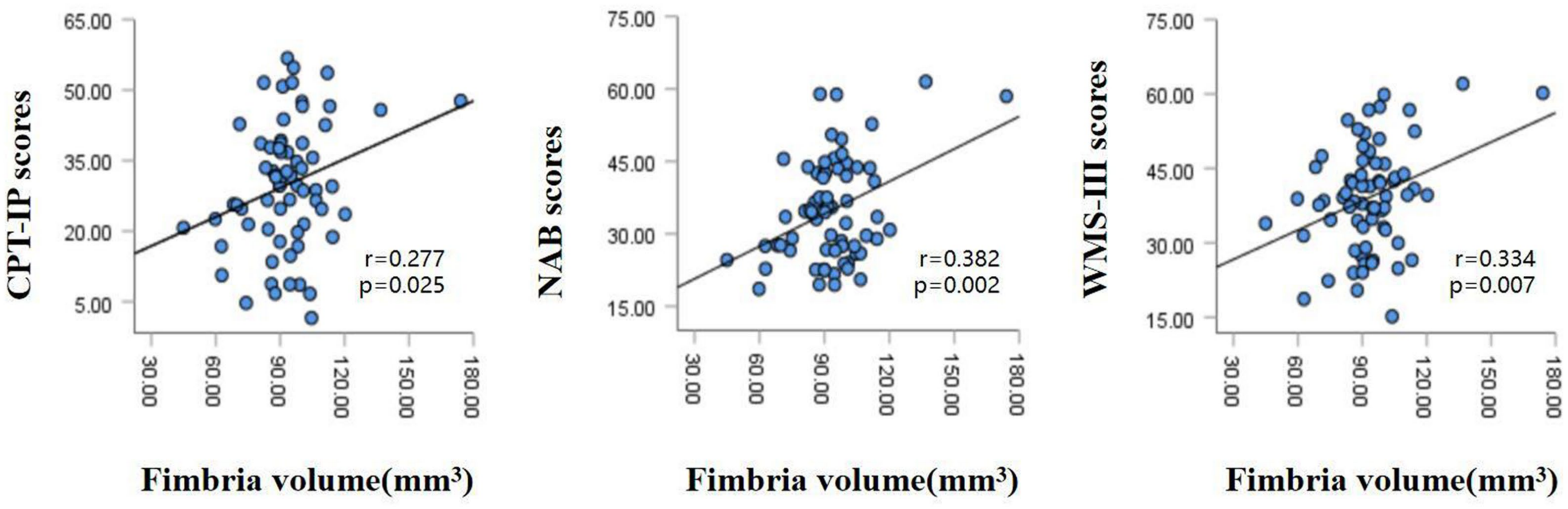
Relationships between hippocampal fimbria volume and CPT-IP scores, WMS-III scores and NAB scores in patients with schizophrenia, controlling for age and sex. CPT-IP, Continuous Performance Test-Identical Pairs; WMS-III, Wechsler Memory Scale, 3rd edition; NAB, Neuropsychological Assessment Battery.

The partial correlation analysis controlling for age and sex showed that within the patient group, the fimbria volume was significantly positively associated with CPT-IP scores (*r* = 0.277, *p* = 0.025), WMS-III scores (*r* = 0.334, *p* = 0.007) and NAB scores (*r* = 0.382, *p* = 0.002; Figure 2). There were no significant relationships between other subfield volumes and cognitive function (Supplementary Table S1).Within the patient group, serum levels of SOD showed a significantly positive correlation with fimbria volume (*r* = 0.360, *p* = 0.003); serum levels of folate showed a significantly positive correlation with hippocampal fissure volume (*r* = 0.286, *p* = 0.021); serum levels of UA showed significantly positive correlations with whole hippocampal volume (*r* = 0.395, *p* = 0.001), hippocampal tail volume (*r* = 0.318, p = 0.01), presubiculum volume (*r* = 0.311, *p* = 0.012), subiculum volume (*r* = 0.311, *p* = 0.012), CA1 volume (*r* = 0.309, *p* = 0.012), CA3 volume (*r* = 0.336, *p* = 0.006), CA4 volume (*r* = 0.379, *p* = 0.002), molecular layer volume (*r* = 0.350, *p* = 0.004) and GC-ML-DG volume (*r* = 0.358, *p* = 0.003; Table 3). However, after multiple comparison corrections, only the relationship between fimbria volume and NAB scores (*r* = 0.382, *p*FDR = 0.024) and the relationship between SOD and fimbria volume (*r* = 0.360, *p*FDR = 0.036) remained significant.

**Table 3:** Relationships between oxidative stress and hippocampal subfield volumes (mm^3^) in patients with schizophrenia controlling for age and sex.

	Folate(ng/ml)		SOD(U/ml)		UA(μmol/L)		Hcy(μmol/L)	
	*r*	*p*	*r*	*p*	*r*	*p*	*r*	*p*
Whole hippocampus	−0.014	0.912	0.142	0.258	0.395	0.001**	0.151	0.229
Hippocampal tail	−0.132	0.294	0.036	0.777	0.318	0.01*	0.175	0.164
Presubiculum	0.023	0.858	0.109	0.386	0.311	0.012*	0.146	0.245
Parasubiculum	−0.106	0.399	−0.022	0.864	0.158	0.21	0.189	0.131
Subiculum	0.146	0.246	0.152	0.228	0.311	0.012*	0.064	0.613
Hippocampal fissure	0.286	0.021*	0.058	0.647	0.137	0.275	−0.052	0.679
CA1	0.018	0.888	0.156	0.214	0.309	0.012*	0.097	0.444
CA3	−0.01	0.934	0.031	0.806	0.336	0.006**	0.03	0.81
CA4	−0.045	0.722	0.099	0.431	0.379	0.002**	0.164	0.193
Molecular layer	0.007	0.957	0.122	0.332	0.35	0.004**	0.117	0.352
GC-ML-DG	−0.056	0.659	0.141	0.262	0.358	0.003**	0.161	0.201
Fimbria	0.072	0.568	0.36	0.003**	0.222	0.075	0.014	0.911
HATA	−0.097	0.44	0.126	0.315	0.226	0.071	0.225	0.072

CA, cornu ammonis; GC-ML-DG, granule cells in the molecular layer of the dentate gyrus; HATA, hippocampal-amygdaloid transition area; SOD, superoxide dismutase; UA, uric acid; Hcy, homocysteine. * *p* < 0.05, **p < 0.01.

### Association between oxidative stress and cognitive function was mediated by hippocampal fimbria volume

**Figure 3:**
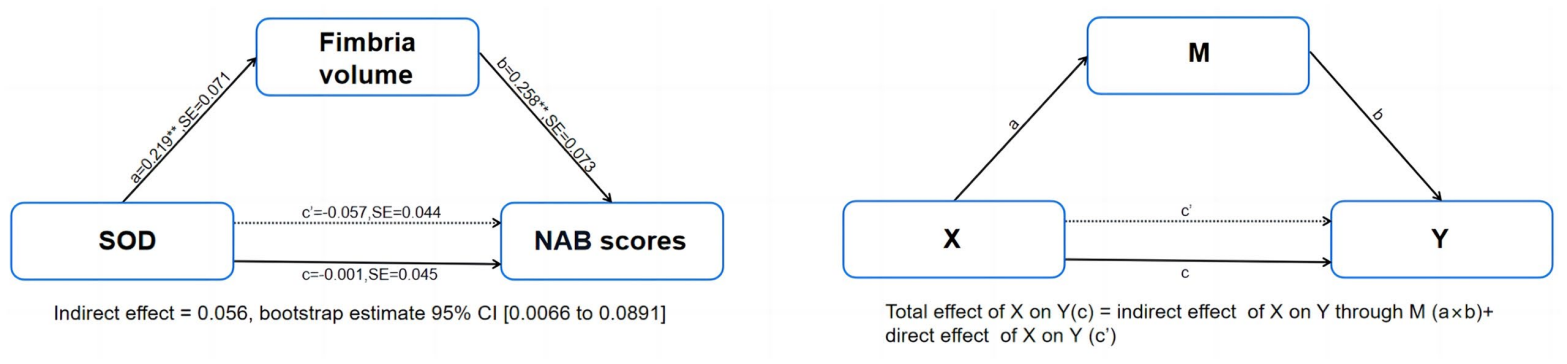
Mediation model of the interrelationships among serum levels of SOD, fimbria volume and NAB scores in patients with schizophrenia, controlling for age and sex. NAB, Neuropsychological Assessment Battery; SE, stand error. **p < 0.01.

Mediation analyzes controlling for age and sex showed that serum levels of SOD in patients with SCZ had significant indirect effects on NAB scores via fimbria volume [indirect effect = 0.0565, 95% CI from the bootstrap test excluding zero (0.0066 to 0.0891)] and had no significant direct effect on NAB scores (direct effect = −0.0572, 95%) CI from the bootstrap test including zero (0.2005 to−0.1455; Figure 3). The mediation effects were not significant for CPT-IP scores and WMS-III scores (Supplementary Figure S1).

## Discussion

This was the first study to show the mediation model between oxidative stress and cognitive impairments via hippocampal subfield volumes. Our findings contained the following points: (1) We found that hippocampal volume deficits were regionally selective in the patient group. (2) The fimbria volume was associated with CPT-IP scores, WMS-III scores and NAB scores in SCZ. (3) Decreased serum levels of folate and SOD and increased Hcy levels were found in SCZ; hippocampal volume deficits in certain regions were found to be associated with folate, SOD and UA levels (4). Oxidative stress impaired cognitive function by affecting hippocampal fimbria volume in the patient group.

We found significantly smaller CA1, molecular layer, GC-ML-DG and fimbria volume in SCZ patients. Previous studies showed significant volume reduction in the bilateral hippocampus and selected hippocampal subfields in patients with first-episode SCZ (Baglivo et al., 2018; McHugo et al., 2018), while more hippocampal subregion defects were reported in chronic patients (Hu et al., 2020). However, the hippocampal reduction in specific subregions in first-episode SCZ was inconsistent. One study found hippocampal volume defects in early psychosis only existed in the CA composite region, including CA1, CA3, subiculum and the molecular layer (McHugo et al., 2018), while another study found decreased subregions, including CA1, CA3, CA4, and the granule cell layer (GCL) and molecular layer (Baglivo et al., 2018). It is worth noting that patients in previous studies were mostly treated with medication. Compared with previous studies, we found different subregional defects, especially in the hippocampal fimbria. This is probably because some studies had not specifically investigated this subregion. In short, we found hippocampal and subregional volume defects at the time of the first psychotic episode, but the specific subregions differed from those identified in previous studies. The discrepancy in results may have been due to inconsistencies in hippocampal subregion definitions, disease duration, antipsychotic treatment, and other environmental factors.

Our data showed lower serum levels of folate and SOD and higher serum levels of Hcy in drug-naïve SCZ patients, and there was no difference in serum levels of UA between the SCZ patients and HCs. These results were in line with our previous findings (Tao et al., 2020). Patients with SCZ have been reported to suffer from an oxidative imbalance between the production of free radicals and antioxidant defense systems. The previous findings of increased oxidative stress in brain tissue, cerebrospinal fluid and peripheral blood were consistent in the SCZ patients (Chowdari et al., 2011; Wu et al., 2013; Guan et al., 2019). High levels of Hcy can increase the intracellular concentration of free radicals, which can damage neuronal membranes and further impair brain function (Mahadik and Mukherjee, 1996; Tyagi et al., 2009). To eliminate the harmful effects of free radicals, SOD can convert superoxide to hydrogen peroxide, which is further decomposed to oxygen and water by catalase (Wu et al., 2013). As a member of the B vitamin family, folate is involved in Hcy metabolism, and it shows powerful antioxidant activity (Mitra et al., 2017). An animal study showed that folate deficiency can cause increased Hcy levels and reduced total antioxidant capacity and that folate supplementation can ameliorate this adverse effect (Úbeda et al., 2020). Moreover, folate deficiency and excessive Hcy levels can increase intracellular calcium. Accumulated calcium can promote the production of free radicals and aggravate nerve cell damage (Bondy and LeBel, 1993; Mattson and Shea, 2003).

We found positive associations between the fimbria volume and cognitive domains, including CPT-IP, WMS-III and NAB in the patient group. In previous studies that explored volumetric changes of hippocampal subregions in SCZ, the hippocampal fimbria was an undervalued region. Compared to previous studies, we used a newer version of FreeSurfer to improve the accuracy of hippocampal subregion segmentation. The fimbria-fornix (FF) bundle is the primary fiber pathway for septal cholinergic neurons projecting to the hippocampus. The fornix constitutes the major hippocampal input and output pathway, supporting cognitive function. It is a primary axonal tract that connects the hippocampus to several subcortical structures. For example, the hippocampus projects to the prefrontal cortex via the fornix (Godsil et al., 2013). The fornix begins as the fimbria, and then becomes a detached bundle to form the fornical crus. The two crura join beneath the splenium of the corpus collosum to form the body of the fornix. These fibers descend into the forebrain and become the columns of the fornix, which divide around the anterior commissure, ending in the septal nuclei and predominantly the mammillary bodies (Catani et al., 2002; Douet and Chang, 2015). Damage to the hippocampal fimbria can disrupt the cholinergic system and cause spatial orientation difficulties and memory deficits in animals (Cassel et al., 1997). Smaller hippocampal fimbria volume was associated with poorer cognitive function in patients with Alzheimer’s disease (Evans et al., 2018; Xu et al., 2022). This may be related to its anatomical connections and functional pathways. A reduction in the volume of the hippocampal fimbria could damage the FF, and the integrity of the FF is important to maintain the memory function of the hippocampus (Nilsson et al., 1987).

The present study also found that peripheral blood indicators of oxidative stress were correlated with hippocampal subregion volumes in SCZ patients. To the best of our knowledge, this is the first study to show links between peripheral markers of oxidative stress and hippocampal subregion volumes. The results of our study suggest that increased oxidative stress could be a promising peripheral biomarker of SCZ and indicate central pathophysiological alterations. The hippocampus is highly susceptible to oxidative damage (Fukui et al., 2002; Onodera et al., 2003; Sato et al., 2010). The potential mechanisms involved in the correlation between oxidative stress and hippocampal subregions remain poorly explained. The blood–brain barrier (BBB) is vulnerable to oxidative stress (Banks and Rhea, 2021). Elevated oxidative stress was observed in peripheral blood and brain tissue in SCZ patients (Wu et al., 2013). We can infer that peripheral oxidative stress reflects central oxidative stress to some extent. Oxidative stress has been related to impaired neurogenesis, dendrite atrophy, and synaptic loss (Huang et al., 2012; Manji et al., 2012; Zou et al., 2012). Central oxidative stress may contribute to impaired neurogenesis and reduced neuronal numbers through the disruption of neuronal membranes (Medina-Hernández et al., 2007; Huang et al., 2012; Zou et al., 2012). This damage ultimately results in measurable hippocampal volume defects. One study found that the oxidative stress pathway started in CA3 area, progressed to CA1 area, and then continued to other hippocampal and cortical areas in Alzheimer’s disease (Cruz-Sánchez et al., 2010). Oxidative stress is one of the important mechanisms of insulin resistance (Houstis et al., 2006). Insulin receptors are highly expressed in the hippocampus. Insulin signaling can promote the formation of hippocampal dendritic spines and synapses as well as neurogenesis (Ferrario and Reagan, 2018; Chavoshinezhad et al., 2021). When central insulin resistance occurs, hippocampal integrity is consequently compromised. Oxidative stress is also related to inflammation in organisms. Oxidative stress can stimulate the activation of inflammatory cells (Morris et al., 2022). In turn, inflammation leads to increased ROS production and reduced intracellular antioxidant capacity (Mittal et al., 2014). Nuclear factor-kB (NF-kB) plays an important role in the relationship between inflammation and oxidative stress. The NF-kB activation triggered by superoxide can boost the release of inflammatory factors, such as IL-1, IL-6 and TNF-α (Flohé et al., 1997; Liu et al., 2017). Higher levels of IL-6 and TNF-α were found to be associated with smaller hippocampal volumes (Sudheimer et al., 2014; Miller et al., 2021).

Regarding the relationships among oxidative stress, hippocampal subfield volumes and cognitive impairments in SCZ, we found that serum levels of SOD had significant indirect effects on NAB scores, which were entirely mediated by the fimbria volume. We did not find significant direct and total effects. This may be because cognitive function is related to multiple imaging metrics rather than just one specific region of the hippocampal subfields. Abnormalities in brain structure were commonly observed in SCZ. Several studies examined the relationship between abnormal brain structure and cognition in patients with SCZ. For example, attentional impairments were associated with altered temporal and frontal cortical thickness (Edgar et al., 2012). Executive cognitive functions were associated with the prefrontal cortex and thalamus (Giraldo-Chica et al., 2018).

This study provides evidence of the relationship between oxidative stress indicators and hippocampal subfield volumes in SCZ and preliminary support for the role of hippocampal fimbria volume in mediating the relationship between oxidative stress and cognitive function. In conclusion, our study showed that oxidative stress, a reduction in hippocampal subfield volumes and cognitive impairments occur in early SCZ. Oxidative stress impairs cognitive function by affecting hippocampal subfield volumes. The mediation model provides a useful framework for the relationship among oxidative stress, hippocampal subfield volumes and cognitive impairments because the effects are transmitted from oxidative stress to the brain and cognition, not the opposite. In addition, we used a cross-sectional design for this study. Future longitudinal studies with larger sample sizes are needed to clarify the causal relationship among oxidative stress, hippocampal subfield volumes and cognitive impairments.

## Data availability statement

The datasets presented in this article are not readily available because the private information of participants. Requests to access the datasets should be directed to RJ, rufeijia@163.com.

## Ethics statement

The studies involving human participants were reviewed and approved by the Ethics Committee of the First Affiliated Hospital of Zhengzhou University. The patients/participants provided their written informed consent to participate in this study. Written informed consent was obtained from the individual(s) for the publication of any potentially identifiable images or data included in this article.

## Author contributions

All authors listed have made a substantial, direct, and intellectual contribution to the work and approved it for publication.

## Funding

This study was supported by the National Natural Science Foundation of China (nos. 81971253 and U21A20367 to S-XQ, nos. 82201657 to Y-XX), Scientific Research and Innovation Team of The First Affiliated Hospital of Zhengzhou University (no. ZYCXTD2023015 to S-XQ), Project for Science and Technology Innovation Teams in Universities of Henan Province (nos. 21IRTSTHN027 to S-XQ), Zhong yuan Technological Innovation leading Talents (204200510019 to S-XQ), Provincial and Ministry Co-construction Youth Funds of Henan Provincial Health Commission (no. SBGJ202003029 to L-X) and the Project of Science and Technology of Henan (no. LHGJ20220310 to Y-XX).

## Conflict of interest

The authors declare that the research was conducted in the absence of any commercial or financial relationships that could be construed as a potential conflict of interest.

## Publisher’s note

All claims expressed in this article are solely those of the authors and do not necessarily represent those of their affiliated organizations, or those of the publisher, the editors and the reviewers. Any product that may be evaluated in this article, or claim that may be made by its manufacturer, is not guaranteed or endorsed by the publisher.

## Footnotes

^1^ http://surfer.nmr.mgh.harvard.edu
