# Supplementary material for: Oxidative stress impairs cognitive function by affecting hippocampal fimbria volume in drug-naïve, first-episode schizophrenia
Source: Front Neurosci. 2023 Apr 17;17:1153439. doi: 10.3389/fnins.2023.1153439 (PMC10149877; doi:10.3389/fnins.2023.1153439)
Supplement: Supplementary file 1 [file Table_1.docx]

**SUPPLEMENTARY TABLE1 |** Relationships between hippocampal subfield volumes and cognitive function in patients with schizophrenia, controlling for age and sex.

|  | SOP | | CPT-IP | | WMS-Ⅲ | | HVLT-R | | BVMT-R | | NAB | | MSCEIT | |
| --- | --- | --- | --- | --- | --- | --- | --- | --- | --- | --- | --- | --- | --- | --- |
|  | r | P | r | P | r | P | r | P | r | P | r | P | r | P |
| Whole hippocampus | -0.042 | 0.74 | 0.071 | 0.576 | 0.135 | 0.285 | 0.052 | 0.678 | 0.078 | 0.539 | 0.056 | 0.657 | 0.002 | 0.985 |
| Hippocampal tail | -0.123 | 0.331 | -0.026 | 0.839 | 0.006 | 0.959 | -0.01 | 0.939 | -0.024 | 0.852 | -0.129 | 0.304 | 0.128 | 0.31 |
| Presubiculum | -0.044 | 0.729 | 0.087 | 0.49 | 0.129 | 0.305 | 0.054 | 0.671 | 0.088 | 0.486 | 0.144 | 0.252 | -0.05 | 0.694 |
| Parasubiculum | -0.167 | 0.183 | -0.097 | 0.443 | -0.14 | 0.267 | -0.028 | 0.823 | -0.006 | 0.048 | 0.067 | 0.595 | -0.192 | 0.126 |
| Subiculum | 0.061 | 0.628 | 0.165 | 0.19 | 0.156 | 0.215 | 0.179 | 0.153 | 0.185 | 0.14 | 0.162 | 0.197 | -0.01 | 0.938 |
| Hippocampal fissure | 0.047 | 0.71 | 0.009 | 0.946 | 0.144 | 0.252 | 0.164 | 0.19 | 0.156 | 0.214 | 0.067 | 0.597 | -0.045 | 0.725 |
| CA1 | 0.041 | 0.744 | 0.11 | 0.384 | 0.179 | 0.153 | 0.01 | 0.934 | 0.066 | 0.599 | 0.108 | 0.391 | -0.004 | 0.977 |
| CA3 | -0.086 | 0.493 | -0.071 | 0.576 | 0.069 | 0.585 | 0.00 | 0.997 | 0.026 | 0.835 | -0.093 | 0.461 | -0.077 | 0.543 |
| CA4 | -0.108 | 0.391 | -0.015 | 0.908 | 0.087 | 0.491 | 0.039 | 0.757 | 0.048 | 0.707 | -0.027 | 0.831 | -0.039 | 0.76 |
| Molecular layer | -0.015 | 0.904 | 0.084 | 0.507 | 0.126 | 0.318 | 0.046 | 0.717 | 0.101 | 0.425 | 0.066 | 0.599 | -0.001 | 0.992 |
| GC-ML-DG | -0.095 | 0.454 | 0.004 | 0.972 | 0.105 | 0.404 | 0.039 | 0.759 | 0.034 | 0.786 | -0.015 | 0.907 | -0.024 | 0.849 |
| Fimbria | 0.201 | 0.109 | 0.277 | 0.025* | 0.334 | 0.007** | 0.115 | 0.361 | 0.119 | 0.347 | 0.382 | 0.002** | 0.027 | 0.834 |
| HATA | -0.122 | 0.332 | 0.024 | 0.852 | 0.038 | 0.766 | 0.007 | 0.955 | -0.026 | 0.839 | -0.045 | 0.72 | -0.025 | 0.843 |

CA, cornu ammonis; GC-ML-DG, granule cells in the molecular layer of the dentate gyrus; HATA, hippocampal-amygdaloid transition area; SOP, speed of processing; CPT-IP, Continuous Performance Test-Identical Pairs; WMS-III, Wechsler Memory Scale, 3rd edition; HVLT-R, Hopkins Verbal Learning Test-Revised; BVMT-R, Brief Visuospatial Memory Test-Revised; NAB, Neuropsychological Assessment Battery; MSCEIT, Mayer-Salovey-Caruso Emotional Intelligence Test. *p < 0.05; **p < 0.01.
