# Supplementary figures and images for: Oxidative stress impairs cognitive function by affecting hippocampal fimbria volume in drug-naïve, first-episode schizophrenia
Source: Front Neurosci. 2023 Apr 17;17:1153439. doi: 10.3389/fnins.2023.1153439 (PMC10149877; doi:10.3389/fnins.2023.1153439)

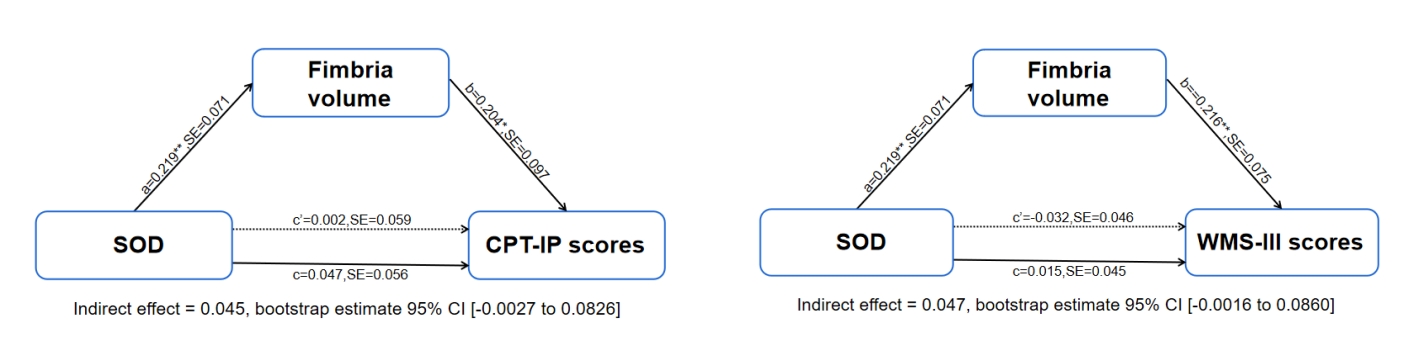

Supplement: Supplementary file 2 [file Image_1.jpeg]
